# High prevalence of previously unknown heart failure and left ventricular dysfunction in patients with type 2 diabetes

**DOI:** 10.1007/s00125-012-2579-0

**Published:** 2012-05-18

**Authors:** L. J. M. Boonman-de Winter, F. H. Rutten, M. J. M. Cramer, M. J. Landman, A. H. Liem, G. E. H. M. Rutten, A. W. Hoes

**Affiliations:** 1 Julius Center for Health Sciences and Primary Care, University Medical Center Utrecht, Utrecht, the Netherlands; 2 Department of Scientific Research, Center for Diagnostic Support in Primary Care (SHL-Groep), Bredaseweg 165, 4872 LA Etten-Leur, the Netherlands; 3 Department of Cardiology, Heart-Lung Center, University Medical Center Utrecht, Utrecht, the Netherlands; 4 Meander Hospital, Amersfoort, the Netherlands; 5 Department of Cardiology, Admiraal de Ruyter Hospital, Goes, the Netherlands; 6 Present Address: Van Hardenbroeklaan 21, 3832CK Leusden, the Netherlands; 7 Present Address: Department of Cardiology, Sint Franciscus Hospital, Rotterdam, the Netherlands

**Keywords:** Diagnosis, Echocardiography, Epidemiology, Heart failure, Left ventricular dysfunction, Prevalence, Type 2 diabetes

## Abstract

**Aims/hypothesis:**

The aim of this study was to assess the prevalence of (unknown) heart failure and left ventricular dysfunction in older patients with type 2 diabetes.

**Methods:**

In total, 605 patients aged 60 years or over with type 2 diabetes in the south west of the Netherlands participated in this cross-sectional study (response rate 48.7%), including 24 with a cardiologist-confirmed diagnosis of heart failure. Between February 2009 and March 2010, the patients without known heart failure underwent a standardised diagnostic work-up, including medical history, physical examination, ECG and echocardiography. An expert panel used the criteria of the European Society of Cardiology to diagnose heart failure.

**Results:**

Of the 581 patients studied, 161 (27.7%; 95% CI 24.1%, 31.4%) were found to have previously unknown heart failure: 28 (4.8%; 95% CI 3.1%, 6.6%) with reduced ejection fraction, and 133 (22.9%; 95% CI 19.5%, 26.3%) with preserved ejection fraction. The prevalence of heart failure increased steeply with age. Heart failure with preserved ejection fraction was more common in women. Left ventricular dysfunction was diagnosed in 150 patients (25.8%; 95% CI 22.3%, 29.4%); 146 (25.1%; 95% CI 21.6%, 28.7%) had diastolic dysfunction.

**Conclusions/interpretation:**

This is the first epidemiological study that provides exact prevalence estimates of (previously unknown) heart failure and left ventricular dysfunction in a representative sample of patients with type 2 diabetes. Previously unknown heart failure and left ventricular dysfunction are highly prevalent. Physicians should pay special attention to ‘unmasking’ these patients.

## Introduction

Cardiovascular diseases are of major importance in patients with type 2 diabetes, accounting for up to 80% of the excess mortality in these patients [1]. Processes underlying the excess cardiovascular mortality risk include coronary atherosclerosis, generalised microvascular disease and autonomic neuropathy [1]. In addition, myocardial abnormalities (‘diabetic cardiomyopathy’) and heart failure seem to play a role [2, 3]. In general, underdiagnosis of heart failure is common [4]; a prevalence of unrecognised heart failure of up to 20.5% has been reported in specific patient groups, such as patients with chronic obstructive pulmonary disease [4, 5]. Previously reported heart failure prevalence estimates in patients with type 2 diabetes were based on medical records or heart failure scores, lacking echocardiography in all patients. Reported prevalence ranged from 9.5% to 22.3% [6–9], and the incidence of heart failure in patients with type 2 diabetes was about 2.5 times that in people without diabetes [10]. In one study, echocardiography was used to diagnose heart failure with reduced ejection fraction (HFREF), resulting in a prevalence of 7.7%, but diastolic dysfunction and heart failure with preserved ejection fraction (HFPEF) was not assessed [11].

To our knowledge, exact prevalence estimates of (unrecognised) heart failure, with and without reduced ejection fraction, and systolic and diastolic dysfunction in a representative sample of all older patients with type 2 diabetes are lacking. We therefore assessed this prevalence in patients aged 60 years and older with type 2 diabetes, all undergoing echocardiography.

## Methods

### Participants

The study was conducted between February 2009 and March 2010, in the province of Zeeland, in the south west of the Netherlands. We were able to invite a representative group of patients with type 2 diabetes, at least for Western Europe, because all patients with type 2 diabetes in this region are enrolled in the Diabetes Care programme of the Center for Diagnostic Support in Primary Care (SHL), including those (co-)treated by hospital specialists (~50,000 patients during the period of this study). Of all the patients with type 2 diabetes from the participating physicians in this study, 1,243 were 60 years or older, and were invited.

All participants gave written informed consent, and the institutional review board of the University Medical Center Utrecht and the Admiraal de Ruyter Hospital in Goes, the Netherlands approved the study protocol. The protocol of the study has been published previously [12] and the study is registered at www.ccmo.nl, NL2271704108.

### Measurements

The patients without a cardiologist-confirmed diagnosis of heart failure (i.e. including echocardiographic evidence of left ventricular dysfunction) underwent a standardised diagnostic assessment, which was executed in the cardiology outpatient department of the Admiraal de Ruyter Hospital in Goes. Information on duration of diabetes, smoking habits and comorbidities was obtained from the patients and the registry. Patients were asked to bring their medication packages so that current drug treatment could be checked. The presence of angina pectoris and shortness of breath was assessed with the WHO questionnaires [13]. Symptoms and signs were assessed by a trained physician in a standardised manner. A standard 12-lead ECG was recorded and classified according to the Minnesota coding criteria by a single experienced cardiologist, blinded to all other test results. Systolic and diastolic blood pressure was measured once electronically, after 5 min of rest in a supine position. Blood was taken within 2 weeks of the diagnostic assessment, with measurement of serum B-type natriuretic peptide (NT-proBNP), blood glucose, creatinine and HbA_1c_. NT-proBNP was measured with a non-competitive immunoradiometric assay (Roche Diagnostics, Mannheim, Germany).

Echocardiography was performed with a General Electric, Vivid 7 imaging system device (GE Vingmed Ultrasound AS, Horten, Norway) by well-trained and experienced cardiac sonographers. Variables from Doppler analysis, M-mode echocardiography and two-dimensional transthoracic echocardiography were used. Where image quality was adequate, left-ventricular ejection fraction (LVEF) was calculated from the endocardial surface tracings in the apical four-chamber view and two-chamber view, using Simpson’s rule (disc summation method) [14, 15].

LVEF could be assessed in 97.5% of the patients by a quantitative method or the two-dimensional visual estimate method (‘eyeballing’) [16]. The accuracy of eyeballing has been validated previously [17]. Wall motion abnormalities were visually analysed and summarised in a wall motion score [18]. Left ventricular mass was calculated using M-mode measurements and the formula of Devereux and Reichek [19]. Valve regurgitation was graded semi-quantitatively, and, in the case of aortic stenosis, the pressure gradient was measured. Left atrial (LA) volume was assessed by the biplane area–length method from apical four- and two-chamber views [20]. Indexed values were corrected for body surface area. The cut-off values 28 and 34 ml/m^2^ were used for normal and definitely increased LA volume index, respectively [21].

Mitral inflow and pulmonary venous inflow were assessed by means of pulsed-wave Doppler echocardiography. From the mitral inflow profile, the early diastolic mitral flow velocity (E) and atrial contraction (A)-wave velocity and the E-deceleration time were measured, and the early-to-atrial left ventricular filling ratio (E/A) was calculated. The flow velocities of the left or right upper pulmonary vein were recorded, and the ratio of systolic to diastolic forward flow (S/D) was calculated. We measured the peak velocity of the tricuspid regurgitated signal with continuous-wave Doppler and calculated the systolic pulmonary artery pressure with the modified Bernoulli’s equation [22].

Diastolic function was assessed by an approach that integrates Doppler measurements of the mitral inflow and Doppler tissue imaging of the mitral annulus using the early diastolic septal annular velocity (e’) [23]. E’(early diastolic mitral annular velocity) is a measure of the relaxation of the ventricle. We calculated the early filling to early diastolic mitral annular velocity ratio (E/e') as a measure of filling pressures [24].

### Criteria to establish diastolic and systolic dysfunction and heart failure

An E/e' value below 8 was considered normal, and 8–15 indeterminate. An E/e' ≥ 15 was considered abnormal [25], and these patients were classified as having diastolic dysfunction. When E/e' was between 8 and 15 and a septal e' <8 cm/s, a combination of elevated values of the indexed volume of the left atrium, the mitral inflow and pulmonary venous flow were used to classify the presence or absence of diastolic dysfunction. Diastolic function was categorised as normal, impaired relaxation (grade I), pseudo normal filling (grade II) or restrictive filling (grade III) by a combination of age-corrected values of E/A velocity ratio, the E-deceleration time, and S/D (see Table 1) [26]. Patients with E/e' between 8 and 15 and a septal e' <8 cm/s, who had echocardiographic left ventricular hypertrophy or elevated indexed LA volume or S/D < 1 were also classified as having diastolic dysfunction. In patients with atrial fibrillation, we considered an elevated indexed LA volume sufficient to classify as diastolic dysfunction.

**Table 1:** Age-corrected Doppler mitral inflow and pulmonary flow profiles used to classify diastolic function

Variable	Normal	Impaired relaxation (grade I)	Pseudo normal filling (grade II)	Restrictive filling (grade III)
E/A (cm/s)	0.75<E/A<1.5	E/A≤0.75	0.75<E/A<1.5	E/A>1.5
DT (ms)	140<DT<320	≥180	140<DT<320	DT<140
S/D	≥1	≥1	<1	<1

DT, deceleration time

Systolic dysfunction was defined as an LVEF ≤ 45% by echocardiography, and diastolic dysfunction graded as I, II or III in combination with an LVEF > 45%.

To be classified as heart failure, systolic or diastolic dysfunction had to be present in combination with one or more suggestive symptoms (e.g. orthopnoea, paroxysmal nocturnal dyspnoea, fatigue, peripheral oedema, nocturia more than twice a night) and one or more signs indicative of heart failure (e.g. peripheral or pulmonary fluid retention or raised jugular venous pressure). In patients who used diuretics, signs of volume overload were not obligatory to classify the presence of heart failure.

Presence or absence of dysfunction and heart failure was determined by an expert panel consisting of two cardiologists (MJC and MJL) and one general practitioner with special interest in heart failure (FHR). The panel was guided by the diagnostic principles of the most recent guidelines for heart failure of the European Society of Cardiology (ESC) [27]. All available results from the diagnostic assessment except for the NT-proBNP results were used. This was done because we wanted to assess the diagnostic value of NT-proBNP separately. The expert panel also established the most likely cause of heart failure based on the diagnostic assessment, including ECG and echocardiography. In the case of no consensus, the majority decision was used.

A random sample of 63 (10.7%) patients was reclassified by the expert panel blinded to the original classification. In five cases, the diagnosis ‘presence or absence of heart failure’ did not correspond to the original diagnosis (κ 0.82, SD 0.08). Incongruent cases most often occurred between diastolic dysfunction and diastolic heart failure.

### Data analyses

We calculated age- and sex-specific prevalence rates of unrecognised HFREF and HFPEF and systolic and diastolic dysfunction. Overall heart failure prevalence estimates were calculated by including patients who already had a cardiologist-confirmed diagnosis of heart failure at baseline in both the numerator and denominator. Prevalence estimates are given for 5-year age groups and for men and women separately. Binominal confidence intervals (95%) were calculated for overall prevalence rates. Data with a skewed distribution were summarised as medians with IQRs. Data were analysed using SPSS Windows version 16.0 for Windows (SPSS, Chicago, IL, USA).

## Results

### Participants

In total, 605 patients agreed to participate (response rate 48.7%), 581 of them without a cardiologist-confirmed diagnosis of heart failure. The mean age of the 605 responders was 71.5 (SD 7.5) years, and 54.0% (95% CI 50.1%, 58.0%) were male. The mean HbA_1c_ was 6.7% (SD 0.5%) (49.0 [SD 6.1] mmol/mol), creatinine 81.7 (SD 15.9) μmol/l, and modification of diet in renal disease (MDRD) 82.8 (SD 15.4) ml min^−1^ 1.73 m^−2^. The mean age of non-responders was 77.0 (SD 9.1) years, the percentage of men 43.4 (95% CI 39.6, 47.3), HbA_1c_ 6.7% (SD 0.6%) (49.4 [SD 6.8] mmol/mol), creatinine 84.5 (SD 19.7) μmol/l and MDRD 79.0 (SD 17.9) ml min^−1^ 1.73 m^−2^. Table 2 shows the characteristics of the participants previously not known to have heart failure.

**Table 2:** Characteristics of patients with type 2 diabetes aged ≥60 years, previously not known to have heart failure, categorised as newly detected heart failure or no heart failure after the diagnostic assessment

Characteristic	All patients (*n* = 581)	Newly detected heart failure (*n* = 161)	No heart failure (*n* = 420)	*p* value
Mean (SD) age (years)^a^	71.6 (7.4)	74.5 (7.7)	70.5 (6.9)	<0.001
Male	310 (53.4)	77 (47.8)	233 (55.5)	0.098
Median (IQR) duration of diabetes^a^	5.5 (3.0, 15.2)	6.3 (3.3, 10.2)	5.1 (3.0, 10.1)	0.292
Mean (SD) HbA_1c_ (%)	6.7 (0.7)	6.7 (0.7)	6.7 (0.7)	0.519
Mean (SD) HbA_1c_ (mmol/mol)	49.5 (8.0)	49.9 (8.2)	49.4 (7.4)	0.519
Current smoker	80 (13.8)	24 (14.9)	56 (13.3)	0.622
Mean (SD) BMI (kg/m^2^)	28.1 (5.1)	29.7 (6.0)	27.6 (4.5)	<0.001
BMI >30 kg/m^2^	163 (28.1)	63 (39.1)	100 (23.8)	<0.001
Comorbidities^b^				
Ischaemic heart disease^c^	108 (18.6)	50 (31.1)	58 (13.8)	<0.001
Atrial fibrillation	42 (7.2)	20 (12.4)	22 (5.2)	0.003
Stroke/TIA	55 (9.5)	23 (14.3)	32 (7.6)	0.014
Peripheral arterial disease	39 (6.7)	21 (13.0)	18 (4.3)	<0.001
Hypertension	381 (65.6)	119 (73.9)	262 (62.4)	0.009
Asthma/COPD	71 (12.2)	28 (17.4)	43 (10.2)	0.018
Renal dysfunction	26 (4.5)	11 (6.8)	15 (3.6)	0.089
Thyroid disease	43 (7.4)	15 (9.3)	28 (6.7)	0.275
Medication				
Loop diuretics	36 (6.2)	22 (13.7)	14 (3.3)	<0.001
Thiazide diuretics	167 (28.7)	51 (31.7)	116 (27.6)	0.333
ACE-inhibitors/angiotensin receptor blockers	306 (52.7)	102 (63.4)	204 (48.6)	<0.001
Aldosterone antagonist	4 (0.7)	1 (0.6)	3 (0.7)	0.903
β-Blockers	209 (36.0)	84 (52.2)	125 (29.8)	<0.001
Dihydropyridines	30 (5.2)	14 (8.7)	16 (3.8)	0.017
Non-dihydropyridines	65 (11.2)	26 (16.1)	39 (9.3)	0.019
Metformin	302 (52.0)	91 (56.5)	211 (50.2)	0.175
Sulfonylurea derivatives	213 (36.7)	59 (36.6)	154 (36.7)	0.996
Thiazolidinediones	17 (2.9)	4 (2.5)	14 (3.1)	0.696
Insulin	73 (12.6)	23 (14.3)	50 (11.9)	0.438
Symptoms				
Dyspnoea	251 (43.2)	117 (72.7)	134 (31.9)	<0.001
Fatigue	228 (39.2)	104 (64.6)	124 (29.5)	<0.001
Paroxysmal nocturnal dyspnoea or orthopnoea	61 (10.5)	31 (19.3)	30 (7.1)	<0.001
Swollen ankles	161 (27.7)	75 (46.6)	86 (20.5)	<0.001
Nocturia	260 (44.8)	94 (58.4)	166 (39.5)	<0.001
Angina pectoris	48 (8.3)	24 (14.9)	24 (5.7)	<0.001
Palpitations	113 (19.4)	43 (26.7)	70 (16.7)	0.006
Additional tests				
Abnormal ECG^d^	342 (58.9)	128 (79.5)	214 (51.0)	<0.001
NT-proBNP ≥15 pmol/l (125 pg/ml)	183 (31.5)	87 (54.0)	96 (22.9)	<0.001
Median (IQR) NT-proBNP	9.0 (5,18)	16.0 (9, 41)	8 (5, 14)	<0.001
Echocardiographic variables				
Mean (SD) LVEF	60.2 (8.2)	56.9 (9.4)	61.4 (7.4)	<0.001
LVEF >55%	438 (75.4)	107 (66.5)	331 (78.8)	0.002
LVEF 45–55%	117 (20.1)	34 (21.1)	83 (19.8)	0.430
LVEF ≤45%	26 (4.5)	20 (12.4)	6 (1.4)	<0.001
Mean (SD) e'	0.06 (0.02)	0.06 (0.2)	0.07 (0.2)	<0.001
Mean (SD) E/e'	9.5 (3.4)	11.4 (5.0)	8.8 (2.3)	<0.001
Mean (SD) LA volume index (ml/m^2^)	27.7 (9.0)	32.6 (10.3)	25.9 (7.8)	<0.001
Mean (SD) LVED volume index (ml/m^2^)	43.8 (14.9)	47.7 (18.1)	42.4 (13.4)	0.002
Diastolic dysfunction grade I	290 (50.5)	141 (89.8)	149 (35.7)	<0.001
Diastolic dysfunction grade II	13 (2.3)	11 (7.0)	2 (0.5)	<0.001
Diastolic dysfunction grade III	3 (0.5)	3 (1.9)	0 (0.0)	0.005

Values are number (%) unless stated otherwise

^a^At time of investigation

^b^Comorbidities mentioned by the patient during history taking

^c^Prior myocardial infarction, angina pectoris, coronary bypass grafting or percutaneous intervention

^d^Any abnormality

COPD, chronic obstructive pulmonary disease; LVED, left ventricular end diastolic; TIA, transient ischaemic attack

### Prevalence of heart failure

Of the 581 patients previously not known to have heart failure, 161 were diagnosed with heart failure (prevalence 27.7%; 95% CI 24.1%, 31.4%): 28 (4.8%; 95% CI 3.1%, 6.6%) with HFREF, 133 (22.9% 95% CI 19.5%, 26.3%) with HFPEF, and none with right-sided heart failure. Of those with HFREF, nine had an LVEF ≤ 40%, and 19 an LVEF between 40% and 45%. The overall prevalence of heart failure among the 605 patients was 185 (30.6%; 95% CI 26.9%, 34.2%): 35 (5.8%; 95% CI 3.9%, 7.6%) with HFREF, and 150 (24.8%; 95% CI 21.4%, 28.2%) with HFPEF. In addition, five patients were classified as having possible heart failure by the expert panel: one with HFREF, three with HFPEF, and one with right-sided heart failure. Sex-specific prevalence rates for 5-year age groups of previously unknown HFREF and HFPEF are shown in Table 3. The prevalence of heart failure increased with age, and was overall higher in female than male patients (31.0% vs 24.8%). The prevalence of HFREF was higher in men (6.8%) than women (3.0%).

**Table 3:** Prevalence of previously unknown HFREF and HFPEF stratified by age and sex in patients with type 2 diabetes aged ≥60 years

Age (years)	Male				Female				Total			
	*n*	HFREF	HFPEF	All HF	*n*	HFREF	HFPEF	All HF	*n*	HFREF	HFPEF	All HF
60–64	85	3 (3.5)	14 (16.5)	17 (20.0)	60	1 (1.7)	6 (10.0)	7 (11.7)	145	4 (2.8)	20 (13.8)	24 (16.6)
65–69	64	3 (4.7)	10 (15.6)	13 (20.3)	67	2 (3.0)	13 (19.4)	15 (22.4)	131	5 (3.8)	23 (17.6)	28 (21.4)
70–74	65	4 (6.2)	6 (9.2)	10 (15.4)	51	0 (0)	19 (37.3)	19 (37.3)	116	4 (3.4)	25 (21.6)	29 (25)
75–80	49	3 (6.1)	9 (18.4)	12 (24.5)	44	2 (4.5)	20 (45.5)	22 (50.0)	93	5 (5.3)	29 (31.2)	34 (36.6)
≥80	47	7 (14.9)	18 (38.3)	25 (53.2)	49	3 (6.1)	18 (36.7)	21 (42.9)	96	10 (10.4)	36 (37.5)	46 (47.9)
All ages	310	20 (6.8)	57 (18.4)	77 (24.8)	271	8 (3.0)	76 (28.0)	84 (31.0)	581	28 (4.8)	133 (22.9)	161 (27.7)

Values are number (%)

### Prevalence of heart failure in specific subgroups

The prevalence was 38.7% (95% CI 31.2%, 46.1%) in patients with a BMI ≥ 30 kg/m^2^ and 23.4% (95% CI 19.4%, 27.5%) in those with a BMI <30 kg/m^2^. In patients with dyspnoea, the prevalence was 46.6% (95% CI 40.4%, 52.7%) and 13.3% (95% CI 9.7%, 17.0%) in those without dyspnoea. In patients with fatigue, the prevalence was 45.6% (95% CI 39.1%, 52.0%) and in those without fatigue 16.1% (95% CI 12.3%, 20.0%). In patients treated for hypertension, the prevalence was 31.2% (95% CI 26.6%, 35.9%) and in those not treated for hypertension 21% (95% CI 15.4%, 26.6%).

### Prevalence of left ventricular dysfunction

In addition, left ventricular dysfunction was diagnosed in 150 patients (25.8%; 95% CI 22.3%, 29.4%); nearly all (97.3%) had diastolic dysfunction. Of the 581 patients, 264 (45.4%) had ‘normal’ left ventricular function, although 65 (11.2%) had an LVEF 45–55% (without diastolic dysfunction) and could be considered to have suboptimal systolic left ventricular function. Prevalence rates for diastolic and systolic dysfunction are shown in Table 4. The systolic dysfunction was higher in men (1.3%) than in women (0%).

**Table 4:** Prevalence of previously unknown systolic and diastolic dysfunction stratified by age and sex in patients with type 2 diabetes aged ≥60 years

Age (years)	Male				Female				Total			
	*n*	S-Dys	D-Dys	A-Dys	*n*	S-Dys	D-Dys	A-Dys	*n*	S-Dys	D-Dys	A-Dys
60–64	85	0	28 (32.9)	28 (32.9)	60	0	13 (21.7)	13 (21.7)	145	0	41 (28.3)	41 (28.3)
65–69	64	1 (1.6)	10 (15.6)	11 (17.2)	67	0	20 (29.9)	20 (29.9)	131	1 (0.8)	30 (22.9)	31 (23.6)
70–74	65	2 (3.1)	22 (33.8)	24 (36.9)	51	0	14 (27.5)	14 (27.5)	116	2 (1.7)	36 (31.0)	38 (32.8)
75–80	49	1 (2.0)	15 (30.6)	16 (32.7)	44	0	10 (22.7)	10 (22.7)	93	1 (1.1)	25 (26.9)	21 (28.0)
≥80	47	0	7 (14.9)	7 (14.9)	49	0	7 (14.3)	7 (14.3)	96	0	14 (14.6)	14 (14.6)
All ages	310	4 (1.3)	82 (26.5)	86 (29.0)	271	0	64 (23.6)	64 (23.6)	581	4 (0.7)	146 (25.1)	150 (25.8)

Values are number (%)

A-Dys, all dysfunction; D-Dys, diastolic dysfunction; S-Dys, systolic dysfunction

### Causes of heart failure

According to the panel, hypertension with or without left ventricular hypertrophy was the most common cause (82%) of heart failure, followed by prior myocardial infarction (23%) and other ischaemic heart disease (39.8%) (Table 5). If the duration of diabetes was longer than 5 years, diabetic cardiomyopathy was often considered to be a possible cause (29.8%). Other common causes of heart failure according to the panel were atrial fibrillation (10.6%), valvular disease (23.6%), or any combination of possible causes. Myocardial infarction and other ischaemic heart disease were more common (46.4% and 50.0%, respectively) in the subgroup with HFREF. Most patients with heart failure were classified as New York Heart Association (NYHA) functional class II or III at the time of investigation (Table 5).

**Table 5:** Likely causes of newly detected heart failure and the NYHA class, according to the panel

Possible cause/NYHA class	HFREF (*n* = 28)	HFPEF (*n* = 133)	All HF (*n* = 161)
Prior myocardial infarction	13 (46.4)	24 (18.0)	37 (23.0)
Other ischaemic heart disease^a^	14 (50)	50 (37.6)	64 (39.8)
Hypertension	18 (64.3)	114 (85.7)	132 (82.0)
Hypertension with left ventricular hypertrophy	13 (46.4)	82 (61.7)	95 (59.0)
Atrial fibrillation	2 (7.1)	15 (11.3)	17 (10.6)
Other rhythm and/or conduction disturbances	3 (10.7)	5 (3.8)	8 (5.0)
Valvular disease	8 (28.6)	30 (22.6)	38 (23.6)
Diabetic cardiomyopathy	12 (42.9)	36 (27.1)	48 (29.8)
Chronic obstructive pulmonary disease	5 (17.9)	9 (6.8)	14 (8.7)
Other	3 (10.7)	9 (6.8)	12 (7.5)
NYHA class			
II	22 (78.6)	98 (73.7)	120 (74.5)
III	4 (14.3)	35 (26.3)	39 (24.2)
IV	2 (7.1)	0	2 (1.2)

Values are number (%) unless stated otherwise. The panel could adjudicate more than one possible cause

^a^Angina pectoris, coronary artery bypass grafting or percutaneous coronary intervention

HF, heart failure

## Discussion

Our study in a large representative group of older patients with type 2 diabetes showed that the prevalence of previously unknown heart failure is very high (27.7%), steeply increases with age, and is overall higher in women (31.0%) than men (24.8%). The prevalence is significantly higher in patients with a BMI ≥ 30 kg/m^2^ (38.7% vs 23.4%), in patients with dyspnoea (46.6% vs 13.3%), in patients complaining of fatigue (45.6% vs 16.1%) and treated for hypertension (31.2% vs 21%). The majority (82.6%) of the patients with newly detected heart failure had HFPEF (prevalence 22.9%). Moreover, the prevalence of left ventricular dysfunction (25.8%), mainly diastolic dysfunction (25.1%), was high. Only 264 (45.4%) of all investigated patients had ‘normal’ left ventricular function, including 65 (11.2%) with a suboptimal LVEF of 45–55%.

The high prevalence of previously unknown heart failure could be due to patients with symptoms of heart failure not going to a physician or physicians not asking the patient about these symptoms. A further possibility is that when patients do present with these symptoms, physicians do not recognise heart failure.

Hypertension was presumed to be the most important possible cause of heart failure in our study population, according to the panel. Previous studies have shown that hypertension generates a high population attributable risk of heart failure [28] and diastolic left ventricular dysfunction [29].

The exact reason for the high prevalence of diastolic dysfunction is not known, although it may be related to early stages of so-called ‘diabetic cardiomyopathy’ [30]. Our finding that the majority of the patients had an E/A ratio <1, and 52.3% had an E/A ratio <0.75, supports this idea. A low E/A ratio has been linked to early stages of diabetic cardiomyopathy [29, 31].

Several limitations of our study should be discussed. The relatively high blood pressure values could be the result of the single measurement used in our study. Importantly, this had no influence on the prevalence estimates of the cause of heart failure adjudged by the panel, since the panel used a history of high blood pressure rather than current blood pressure levels. NT-proBNP levels were not available to the panel, to prevent incorporating bias for the diagnostic part of the study that will be performed. This may have had some influence on the panel’s diagnosis, although the effect is likely to be small because, in all participants, a complete echocardiographic assessment, including tissue Doppler imaging measurements, was available. When evaluating the possible causes of heart failure, it is important to consider that the panel judged ‘likely’ causes on the basis of the diagnostic assessment, but without specific and detailed further investigations. Under-rating the importance of ischaemia as the possible cause of heart failure is therefore likely.

One of the strengths of the study is a relatively high response rate (48.7%) compared with other population-based studies involving extensive diagnostic testing [5]. As can be expected, the non-responders were older and probably more fragile than the participants. This may have led to a limited underestimation of the prevalence. Importantly, the clinical applicability of our results is high, because we studied patients who were able and willing to undergo the relevant diagnostic investigations, as in every day clinical practice. Another strength of our study is that participants underwent all the diagnostic tests required to classify heart failure. The use of Doppler tissue imaging allowed us to measure left ventricular relaxation and filling pressures, largely independently of load, in a reproducible and feasible way [24, 25, 27, 32]. Illustrating the good image quality was the availability of LVEF estimations in almost all patients (97.5%). Moreover, we aimed to prevent overdiagnosis of diastolic dysfunction and HFPEF by applying age-adjusted cut-off values and using strict criteria for diastolic dysfunction [24, 25]. The use of consensus diagnosis by an outcome panel is an established method in case an irreproachable reference standard is lacking, as is the case for heart failure [33, 34]. Outcome panels have been successfully used in previous studies on heart failure by our group [35]. In our study, the reproducibility was high (κ 0.82, SD 0.08), and comparable to previous studies [5].

The prevalence of previously unknown heart failure in our study (27.7%) is even higher than reported in patients aged 65 years and older with chronic obstructive pulmonary disease [5]. The overall prevalence of heart failure (30.6%) in our study is about four times higher than expected in people aged 60 years and older in the general population [36]. Several previous studies reported a lower prevalence of heart failure in patients with type 2 diabetes. Bertoni et al [6] reported 22.3% in patients with an average age of 74 years, with the diagnosis of heart failure based on insurance claims data. Two other studies reported a prevalence of 11.8%, with the diagnosis of heart failure based on heart failure scores, without the use of echocardiography [7, 9]. Only Davis et al [11] used echocardiography to diagnose heart failure; however, only the prevalence of HFREF (7.7%) was assessed, and not HFPEF.

The prevalence of left ventricular dysfunction in our study, when we include those diagnosed with heart failure, would be 53.5%: 48.0% diastolic dysfunction and 5.5% systolic dysfunction. In addition, 11.2% had suboptimal systolic ventricular dysfunction (LVEF 45–55%). Henry et al [37] reported high prevalence rates of ventricular dysfunction among older patients with type 2 diabetes, with similar values for diastolic dysfunction (47%) and a higher prevalence of systolic dysfunction (30%). Their definition of systolic dysfunction, namely LVEF ≤ 55% instead of our definition of LVEF ≤ 45%, is the probable cause of this higher prevalence.

Screening of patients with type 2 diabetes should be considered in the light of the high rates of prevalence (27.7%) of previously unknown heart failure in older patients with type 2 diabetes observed in our study. Physicians should be constantly alert for signs and symptoms indicative of heart failure in these patients. In addition, echocardiography and/or ECG or B-type natriuretic peptide measurements could be part of the yearly monitoring. We need to determine which screening strategy is the most efficient. Although evidence on how to optimally treat patients with HFPEF is scarce, there is consensus that at least optimising blood pressure and reducing heart rate in patients with tachycardia is needed, combined with optimal treatment of (cardiac) comorbidities [38, 39]. An annual check for the presence of heart failure in patients with type 2 diabetes is not yet advised in the diabetes guidelines [40]. Our results could form the evidence base for such a recommendation.

## Abbreviations

A: Atrial contraction
E: Early diastolic mitral flow velocity
E′: Early diastolic mitral annular velocity
E/A: Early-to-atrial left ventricular filling ratio
E/e': Early filling to early diastolic mitral annular velocity ratio
ESC: European Society of Cardiology
HFPEF: Heart failure with preserved ejection fraction
HFREF: Heart failure with reduced ejection fraction
LA: Left atrial
LVEF: Left ventricular ejection fraction
MDRD: Modification of diet in renal disease
NT-proBNP: N-terminal pro B-type natriuretic peptide
NYHA: New York Heart Association
S/D: Systolic-to-diastolic pulmonary venous flow ratio
